# Supplementary material for: Spatial interactions modulate tumor growth and immune infiltration
Source: Res Sq. 2024 May 22:rs.3.rs-3962451. Preprint. [Version 1] doi: 10.21203/rs.3.rs-3962451/v1 (PMC11142313; doi:10.21203/rs.3.rs-3962451/v1)
Supplement: 1 [file NIHPPRS3962451V1-supplement-1.pdf]

S6 Supplemental Information

S6.1 Spatial variegation of tumor-immune interactions

We assess the degree of spatial variegation and patterning in tumor and immune cells as a function of sensitivity and specificity in figure S1. Spatial maps are shown at time to tumor regression (TTR; see figure 4), and thus all tumors have identical total size in figure S1A. Differences in spatial patterns of tumor density are caused by differential immune recruitment (specificity) and strength of predation (sensitivity). As the immune specificity narrows (r_{21} reduces to smaller values), immune cell concentration strongly correlates with the tumor's location with a high degree of specificity (figure S1B, right-to-left). This specificity has only a marginal effect on tumors if the range of sensitivity is diffuse (large r_{12} values). As sensitivity narrows, immune predation is more highly targeted and effective, killing tumor cells, especially in the core (figure S1A, bottom-left). A ring-shaped tumor is left, due to proliferating cells on the rim.

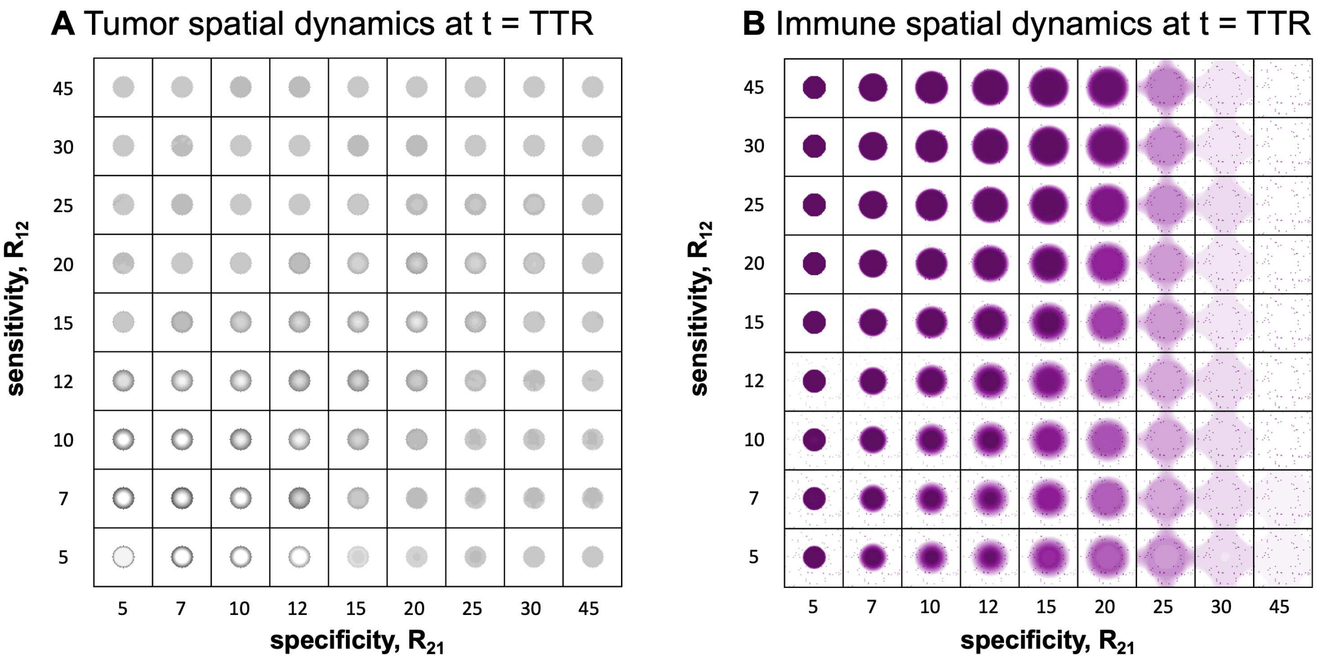

Figure S1. Tumor-immune spatial variegation patterns as a function of interaction kernels Spatial maps are shown at the time to tumor regression (TTR; see figure 4) for a range of tumor-immune sensitivity (r_{12}) and specificity (r_{21}) values. (A) Tumor spatial maps at TTR. (B) Immune spatial maps at TTR. Spatial variegation in tumor density increases as both specificity and sensitivity are increased. As immune specificity narrows (r_{21} is small), immune cell concentration strongly correlates with tumor's location with a high degree of specificity. Unless otherwise noted, parameters used are $\gamma = 5, b = 12, g = 1.5, d = 1, L = 0.08$ (see eqns. 6-7).

S6.2 Stochastic Lenia

The Lenia framework can be extended to a stochastic setting by considering the likelihood of a lattice location $\mathbf{x}$ containing a single cell. Let $\mathbf{A}(\mathbf{x}) \in [0,1]$ be a random variable, such that the probability, P, of a cell potentially being added to an existing lattice location $\mathbf{x}$ at the next time step $t + \Delta t$. This probability is determined by the Binomial distribution (if the lattice location is currently empty), at a rate of $G(\mathbf{U}(\mathbf{x}))\Delta t$.

S6.3 Particle Lenia

Particle Lenia is an extension of Lenia where movement of the particle is determined by the sum of two local gradients representing the energy field, $\mathbf{E}(\mathbf{p}_i)$, and migration field, $\mathbf{M}(\mathbf{p}_i)$. Particle i's location in two-dimensional space is given by $\mathbf{p}_i := \{x, y\}$ and updated according to the following equation:

$$\frac{d\mathbf{p}_i}{dt} = \underbrace{-\nabla\mathbf{E}(\mathbf{p}_i)}_{\texttt{cell adhesion-repulsion}} + \underbrace{\nabla\mathbf{M}(\mathbf{p}_i)}_{\texttt{microenvironment}} = -\frac{\partial\mathbf{E}(\mathbf{p}_i)}{\partial(\mathbf{p}_i)} + \frac{\partial\mathbf{M}(\mathbf{p}_i)}{\partial(\mathbf{p}_i)} \tag{12}$$

Particles (or cells) move against the local gradient of $\mathbf{E}(\mathbf{x})$, the energy field. The cell-cell repulsion and adhesion fields are a function of the potential field, $\mathbf{U}$, such that:

$$\begin{aligned}
\mathbf{E}^t(\mathbf{x}) &= E\left(\mathbf{U}^t(\mathbf{x})\right) && (13)\\
&= E\left(\sum_i \mathbf{K}\left(\mathbf{x} - \mathbf{p}_i^t\right)\right) && (14)\\
&= E\left(\sum_i K\left(\left\|\mathbf{x} - \mathbf{p}_i^t\right\|\right)\right) && (15)
\end{aligned}$$

where $\mathbf{E}^t(\mathbf{x})$ is interpreted as the energy field as a function of the distance from the focal particle. Interacting cells move along the gradient of the energy field, weighted by the interaction kernel as a function of particle-particle distance, and summed over all particles.

S7 Supplemental Video Captions

Figure S2. Supplemental Video S1: Short-range interaction kernels are more robust to Allee effects Spatial maps shown for deterministic Lenia (left) and stochastic Lenia (right) over time.

Figure S3. Supplemental Video S2: tumor-immune spatial variegation patterns as a function of interaction kernels Video of simulations, corresponding to figure S1. Left: Spatial maps shown over time for a range of tumor-immune sensitivity (r_{12}) and specificity (r_{21}) values. Right: Immune spatial maps over time. Spatial variegation in tumor density increases as both specificity and sensitivity are increased. As immune specificity narrows (R_{21} is small), immune cell concentration strongly correlates with tumor's location with a high degree of specificity. Parameters used are $\gamma = 5, b = 12, g = 1.5, d = 1, L = 0.08$ (see eqns. 6-7).

Figure S4. Supplemental Video S3: example immune infiltration simulation for perpendicular and parallel model Video of simulations, corresponding to figure 6. Left: perpendicular model. Right: parallel model.

Figure S5. Supplemental Video S4: example immune infiltration simulation for perpendicular and parallel model Video of simulations, corresponding to figure 6E. Simulations ordered by disease stage from left to right.
